# 3D-evaluation of the maxillary sinus in cone-beam computed tomography

**DOI:** 10.1186/s40729-018-0128-4

**Published:** 2018-06-05

**Authors:** Julia Luz, Dominique Greutmann, Daniel Wiedemeier, Claudio Rostetter, Martin Rücker, Bernd Stadlinger

**Affiliations:** 1 Clinic of Cranio-Maxillofacial and Oral Surgery, University of Zurich, University Hospital Zurich, Zurich, Switzerland; 2 Statistical Services, Center of Dental Medicine, University of Zurich, Zurich, Switzerland

**Keywords:** Cone-beam computed tomography, CBCT, Digital imaging, Maxillary sinus, Volumetric analysis

## Abstract

**Background:**

There are few studies measuring the dimensions of the maxillary sinus, being mostly based on computed tomography imaging and rarely being based on cone-beam computed tomography (CBCT). The aim of this study was to measure the 3D osseous and soft tissue defined volume and surface area of the maxillary sinus. Further, possible associations with patient-specific and sinus-related variables were evaluated.

**Methods:**

A total of 128 maxillary sinuses in 64 patients were analyzed using cone-beam computed tomography data. Surface area and volume of the osseus maxillary sinuses as well as of the remaining pneumatized cavities in cases of obliterated sinuses were calculated by the implant planning software SMOP (Swissmeda AG, Baar, Switzerland). Further, patient-specific general variables such as age, gender, and dentition state as well as sinus-related factors including apical lesions, sinus pathologies, and number of teeth and roots communicating with the maxillary sinus were recorded.

**Results:**

For osseus bordered sinuses, mean surface area was 39.7 cm^2^ and mean volume 17.1 cm^3^. For the remaining pneumatized cavities, mean surface area was 36.4 cm^2^ and mean volume 15 cm^3^. The calculated mean volume of obliterated sinuses (42.2% of all sinuses were obliterated) was 5.1 cm^3^. Further, an association between the obliterated volume and the presence of pathologies was detected. Male patients showed a significantly higher mean osseus volume compared to female patients. No association was apparent between a patient’s age or dentition state and sinus volume, nor for communicating tooth roots and sinus pathologies or unilateral opacity and apical radiolucency. There was also no significant association between bilateral obliterated sinuses and the scan date being in autumn/winter.

**Conclusions:**

The present study showed that the CBCT is suitable for the evaluation of the maxillary sinus. The implant planning software SMOP and its included volume measuring tool are valuable for the analysis of the maxillary sinus, and possible relations with the dentition can be analyzed.

## Background

The precise assessment of the maxillary sinus is important in oral and maxillofacial surgery in cases of traumatology, sinusitis, and dental implantology. After the introduction of cone-beam computed tomography (CBCT) in dental medicine in 1998 [1], the number of clinicians using CBCTs increased constantly. Whereas in 2004, there were only three CBCTs registered in Switzerland, the current number exceeds 600. The CBCT has become an important diagnostic tool in dental medicine due to its high resolution and its possibility to limit imaging to specific areas of interest. Various specialties in dental medicine like oral and maxillofacial surgery and endodontics increasingly utilize CBCT imaging.

In general dentistry, however, panoramic imaging is still more popular than CBCT. The advantages of panoramic imaging are less radiation, less costs, and its suitability for primary diagnostics. The advantages of CBCT on the other hand are a high image quality of high-contrast structures, no geometric distortion, and no superimposition of surrounding anatomical structures [2].

The aim of this study was to examine the suitability of a volume measuring tool, being included in the implant planning software SMOP (Swissmeda AG, Baar, Switzerland), for the measurement of the 3D shape of the maxillary sinus. Next, this tool was used to measure the volume and surface of the maxillary sinuses. To the best of our knowledge, there is currently no study measuring the osseus and mucosal borders in CBCTs on a 3D level for the analysis of volume reduction due to obliteration. By measuring the osseus and mucosal bordered volume (remaining pneumatized cavity), not only the volume of the obliteration could be calculated, but also possible association between sinus obliteration and the dentition state as well as with the presence of periapical radiolucencies and foreign bodies could be analyzed. Further, possible associations between these measured sinus volumes and patient-specific general variables such as age and gender were evaluated.

## Methods

In the present study, 64 CBCT images (128 maxillary sinuses), taken between 1 January 2013 and 31 December 2013 at the Department of Cranio-Maxillofacial and Oral Surgery at the University of Zurich, were included. The inclusion criterion of each CBCT scan was the presence of two complete maxillary sinuses; the osseus borders of both sinuses had to be entirely visible.

The scans were performed using a KaVo 3D eXam CBCT (Biberach, Germany). The settings were 5.0 mA and 120 kV, with a voxel size of 0.125, 0.25, 0.3, or 0.4 mm (exposure time 26.9 s for 0.3/0.4 voxel size or 26.9 s for 0.125/0.25 voxel size). The field of view (FOV) ranged between a height of 10–13.3 cm (patient-adjusted) with a constant diameter of 16 cm.

For the measurement of the maxillary sinus volume, the CBCT images were imported as DICOM files into SMOP, an implant planning software (Swissmeda AG, Baar, Switzerland). This software allows the calculation of volumes (mm^3^) and surfaces (mm^2^) of a 3D object (e.g., maxillary sinus) by interpolating closed curves.

**Fig. 1:**
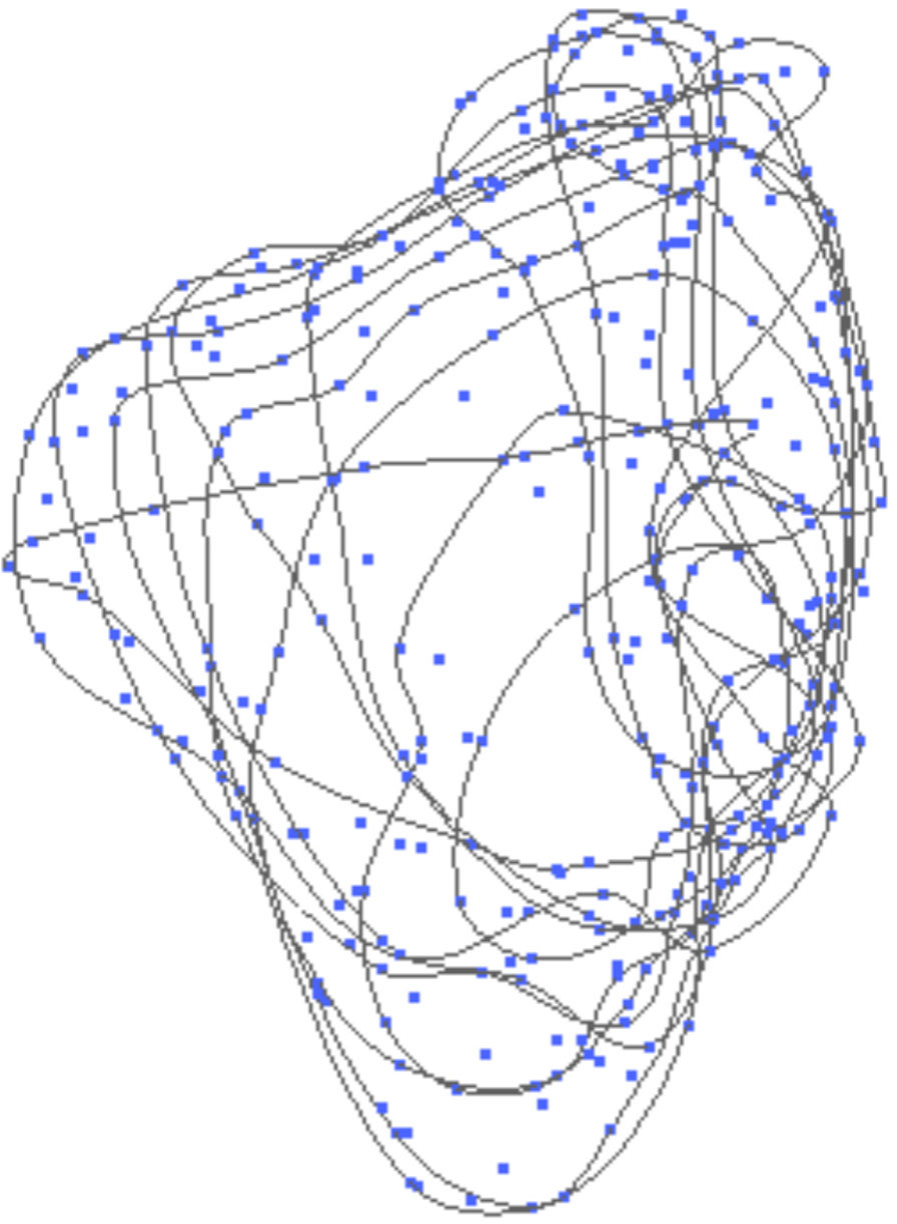
Calculation of the sinus body by interpolating 15–25 curves at a distance of 2 mm, depending upon the size of the maxillary cavity

Using this tool, the volume of each maxillary sinus was calculated by drawing parallel-oriented closed curves in the same coronal plane. Volume measurements were performed in a standardized manner. First, the most posterior and anterior part of each maxillary sinus was defined by placing a curve each in the coronal plane. Next, the space between the two curves was divided into equally sized slices of 2 mm by placing further curves. As a result, a single sinus consisted of 15–25 curves (depending upon the size of the maxillary cavity), having an intercurve distance of 2 mm (Fig. 1).

**Fig. 2:**
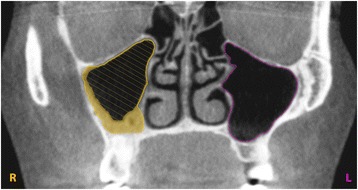
View from the coronal plane. The marked curves define the osseus and mucous boundaries of the maxillary sinuses. The hatched surface illustrates the measured remaining pneumatized cavity of an obliterated sinus and the filled (yellow) surface highlights the calculated obliterated volume

**Fig. 3:**
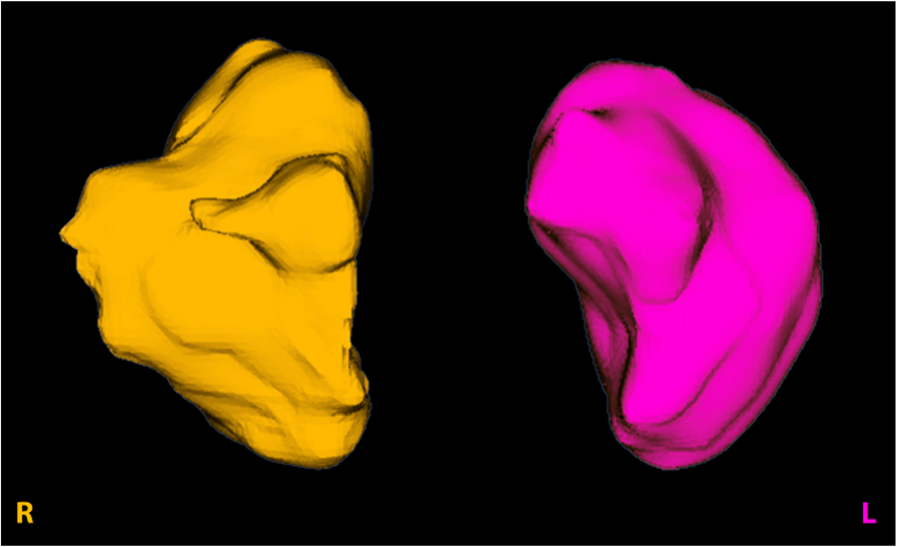
3D view of osseus sinus volumes. Surface area (cm^2^) and volume (cm^3^) were calculated by the software

Some CBCT scans showed sinus cavities that were radiographically partially or fully obliterated indicating a swelling of the mucosa or a sinus pathology. In order to calculate the exact volume of this obliteration, two measurements were performed: first, the sinus volume within the osseus borders was measured by placing the curves on these osseus boundaries. Second, in cases of obliteration, the curves were placed on the mucous borders within the osseus maxillary sinus, measuring the remaining pneumatized cavity. Next, subtracting the two volumes, the obliterated sinus volume was calculated (Figs. 2 and 3).

Patient-specific variables like gender, date of birth, and date of CBCT were recorded. The date of the CBCT image was further divided into either being in autumn/winter (1 January 2013–19 March 2013; 22 September 2013–31 December 2013) or spring/summer (20 March 2013–21 September 2013). The maxillary sinus was classified into obliterated or nonobliterated. It was also documented if there was a unilateral or bilateral obliteration in the CBCT image. Obliterated cavities were further classified using the following radiographic findings: absence of alteration (0), mucosal thickening (1), sinus polyp (2), complete obliteration (3), mucosal thickening and periapical radiolucency (4), foreign body (5), mucosal thickening and foreign body (6), and nonspecific obliteration (7, partial obliteration, not being defined by the previous criteria). Due to the close relationship between the posterior teeth (premolars, molars) and the maxillary sinus, the teeth starting at the first premolar were recorded as either present or missing, along with the presence or absence of endodontic treatment. Additionally, the number of teeth and roots communicating with the maxillary sinus and any apical radiolucency was documented.

### Statistical analysis

The data was primarily analyzed descriptively. The analysis was performed on two different datasets depending on the main question: either on a sinus level consisting of 128 maxillary sinuses or on a patient level consisting of the respective 64 patients. In cases where sinus-level information was associated with patient-level characteristics (presence of pathology vs. obliterated volume, presence of apical radiolucency vs. obliterated volume, presence of pathology vs. number of communicating roots, dentition status vs. osseus sinus volume), one sinus per patient was randomly chosen for the analysis in order to not violate assumptions of independency for the Wilcoxon rank sum and Kruskal-Wallis tests.

For patient-level analysis, the association between a patient’s age and the presence of obliteration was analyzed using logistic regression and patient’s age vs. the mean osseus sinus volume was assessed using linear regression. The Wilcoxon rank sum test was used to investigate if there is an association between the mean osseus sinus volume and gender. Differences between osseus sinus volumes on the left and right side of a patient were assessed with the Wilcoxon signed-rank test. Fisher’s exact test was used to assess possible associations between bilateral obliteration and the date of the CBCT scan (season of the year) as well as between unilateral obliteration and apical radiolucency. The significance level *α* was set to 0.05 for all analyses. Calculations were performed using *R* [3].

## Results

### Sinus-level analysis

**Fig. 4:**
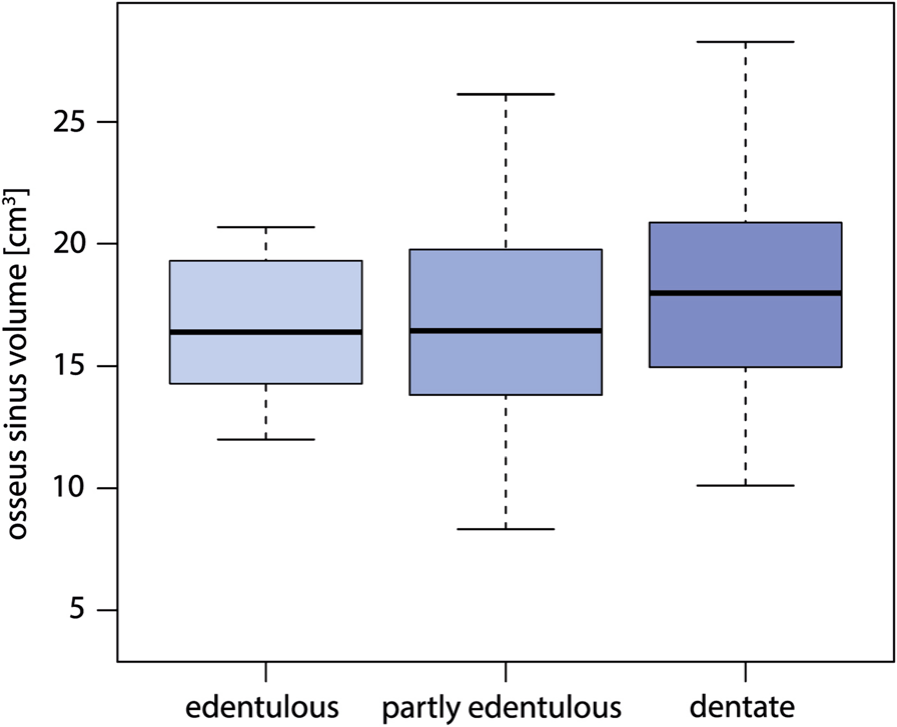
The association between the osseus volume and the dentition. Edentulous, partly edentulous, and dentate patients showed no relevant difference in the size of the osseus sinus volume (*p* = 0.52)

In total, 128 maxillary sinuses were analyzed. The mean surface area was found to be 39.7 cm^2^ and the mean volume 17.1 cm^3^. The mean surface area of the remaining pneumatized cavities of obliterated sinuses was found to be 36.4 cm^2^ and the mean volume 15 cm^3^ (Table 1). 42.2% of all sinuses showed an obliteration, and the mean volume of the obliterated sinuses was 5.1 cm^3^. If there was an obliteration, on average, 27% of the maxillary sinus was obliterated, and overall, the obliterations ranged between 1 and 95%. The dentition state (edentulous, partly edentulous, or dentate posterior region) had no influence on the size of the osseus sinus volume (Fig. 4, *p* = 0.52).

**Table 1:** Mean, median minimum, maximum, and standard deviation of the surface in square centimeter and volume in cubic centimeter of the osseus maxillary sinuses and the remaining pneumatized cavities in cases of obliterated sinuses as well as mean, median, minimum, maximum, and standard deviation of the calculated obliterated sinus volume in cubic centimeter

	Mean	Median	Minimum	Maximum	SD
Osseus sinus surface area (cm^2^)	39.7	39.7	19.1	56.0	7.8
Osseus sinus volume (cm^3^)	17.1	16.8	4.0	28.9	4.8
Remaining pneumatized sinus surface area (cm^2^)	36.4	36.9	15.3	55.8	8.7
Remaining pneumatized sinus volume (cm^3^)	15.0	15.2	0.8	28.0	4.8
Obliterated sinus volume (cm^3^)	5.1	3.5	0.1	26.8	4.7

A total of 73 maxillary sinuses showed unimpaired conditions (57.0%), and 55 showed a pathology (43%). Out of these 55 patients showing a pathology, 30 had a mucosal thickening (23.4%), 17 had a sinus polyp (13.3%), one showed a complete obliteration (0.8%), four had a mucosal thickening and a periapical radiolucency (3.1%), one had a foreign body (0.8%), one had a mucosal thickening and a foreign body (0.8%), and one had a nonspecific opacification (0.8%) (Table 2). Moreover, 20 out of the 55 recorded pathologies were seen in women (36.4%) and 35 in men (63.6%).

**Table 2:** Frequency of pathologies in 128 maxillary sinuses

Frequency of pathologies	*n*	(%)
Absence of alteration	73	(57.0)
Mucosal thickening	30	(23.4)
Sinus polyp	17	(13.3)
Complete opacity	1	(0.8)
Mucosal thickening and periapical radiolucency	4	(3.1)
Foreign body	1	(0.8)
Mucosal thickening and foreign body	1	(0.8)
Nonspecific opacification	1	(0.8)

**Fig. 5:**
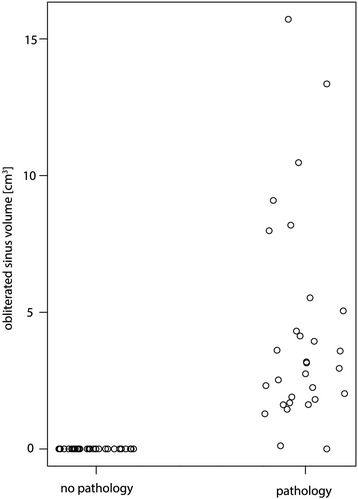
The association between the obliterated volume and sinus pathology. The presence of a pathology significantly increased the obliterated volume of a maxillary sinus (*p* < 0.001). For better visibility, the diagram has been jittered along the *x*-axis

The presence of a pathology significantly (*p* < 0.001) increased the obliterated volume of a maxillary sinus (Fig. 5). Apical radiolucency, on the other hand, did not increase the obliterated volume of the maxillary sinus (*p* = 0.32). There was also no association between the presence of pathology and the number of communicating roots with the maxillary sinus (*p* = 0.62).

### Patient-level analysis

In total, 64 patients were analyzed. Patients had a mean age of 46.2 years. Out of 64 patients, 38 were female (59.4%) and 26 were male (40.6%). Fifty-five patients (85.9%) were dentate or partially dentate and 9 edentulous (14.1%). Fifteen patients (23.4%) had endodontic treatment on at least one tooth in the posterior region of the upper jaw starting from the first premolar. The frequency of teeth communicating with at least one maxillary sinus was 34.4% (22 patients). More than half of the patients (54.7%) had at least one partially or fully obliterated sinus. Out of these 35 patients, 16 had a unilateral obliteration of the maxillary sinus (25%) and 19 had a bilateral obliteration (29.7%). Apical radiolucencies were present in 11 patients (17.2%).

**Fig. 6:**
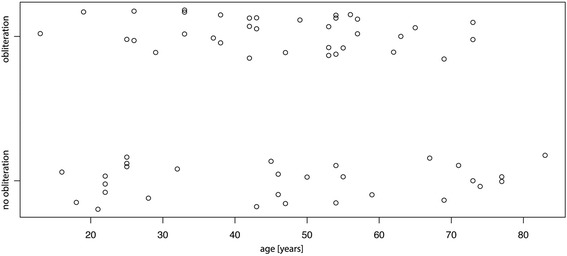
No statistical significant association between a patient’s age and the presence of obliteration of at least one maxillary sinus was found (*p* = 0.92). For better visibility, the diagram has been jittered along the *y*-axis

**Fig. 7:**
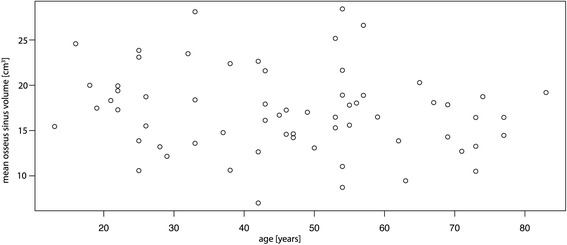
The association between the mean osseus sinus volume and age. No significant association between these parameters was found (*p* = 0.2)

**Fig. 8:**
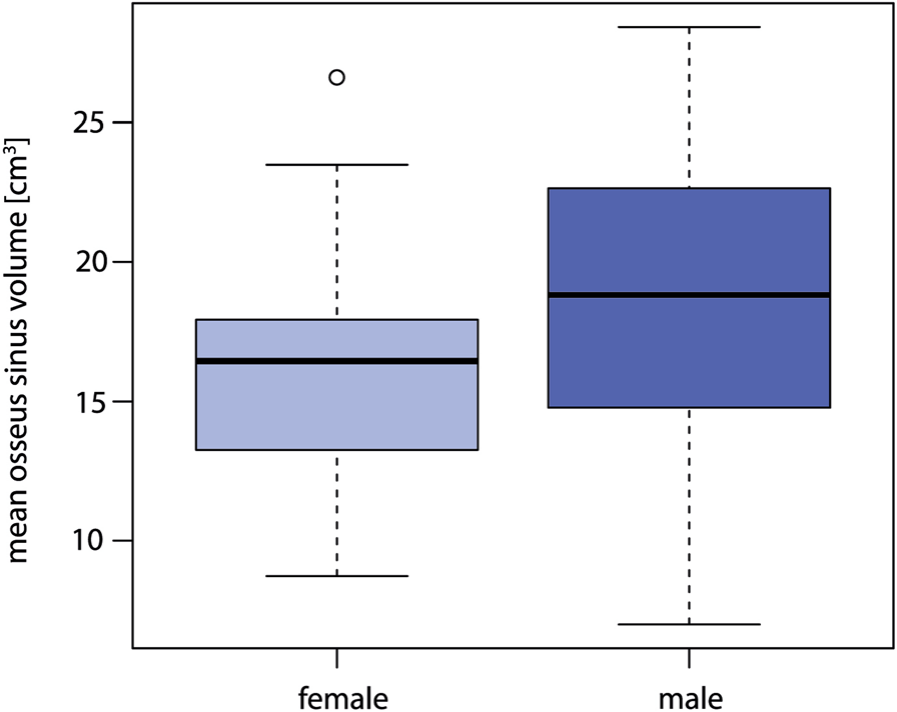
The association between mean osseus sinus volume of the maxillary sinus and gender. Men have a statistically significant higher mean osseus volume than women (*p* = 0.007)

No relationship was observed between a patient’s age and the presence of partial or complete obliteration of at least one maxillary sinus (Fig. 6, *p* = 0.92). Patient’s age and the mean osseus sinus volume were also not associated significantly (Fig. 7, *p* = 0.20). Both maxillary sinuses (osseus borders) of each patient were quite similar in size (mean difference between left and right 0.5 cm^3^), yet statistically significant with slightly larger volumes on the left side (*p* = 0.045). Men were found to have a statistically significant higher mean osseus volume (19.0 cm^3^) than women (15.5 cm^3^) (Fig. 8, *p* = 0.007). No significant association between bilateral obliteration and the date of the CBCT scan (autumn/winter versus spring/summer) could be found (*p* = 0.41). Further, no significant association between unilateral obliterated sinuses and apical radiolucencies was found (*p* = 1).

## Discussion

The aim of this study was to analyze volume parameters of the maxillary sinus based on CBCT data. Further, neighboring anatomical structures and related pathologies were recorded. Overall, the applied volume software used in this study allowed the calculation of the surface area and volume of maxillary sinuses.

In clinics, the radiographic evaluation of the maxillary sinus is obligatory prior to for example sinus floor elevation. Based on this image, the risk for sinus floor elevation and implant placement can be evaluated. The CBCT data can be further used for later implant placement, using guided techniques. CBCT has been proven to be a valuable tool for the analysis of the maxillary sinus as long as the information provided exceeds the radiological risks [4, 5]. Moreover, its accuracy has been proven [6, 7]. Using CBCT images, anatomical structures may be measured in terms of distances as well as volumes.

### Sinus-level analysis

In this study, the measurements were performed using the SMOP volume software. This software was used earlier by another group for the analysis of the 3D shape of nasopalatine duct cysts [8]. The present study measured both the sinus volume within the osseus borders and the remaining pneumatized sinus volume in cases of obliteration. For the osseus bordered sinus, the measured mean sinus volume was 17.1 cm^3^, the minimum 4.0 cm^3^, and the maximum value 28.9 cm^3^. These measurements are quite comparable to the results of other studies [9, 10]. With regard to sinus obliteration, various studies suggest a potential relationship between periapical lesions and mucosal irritation of the maxillary sinus [11–13]. Brook [14] showed that 10–12% of all cases of maxillary sinusitis were caused by teeth. In this study, no association between apical radiolucencies in the upper jaw and sinus obliteration was found. However, it should be mentioned that this study was not designed to observe this relation. In a study, analyzing this association, Nunes et al. [15] selected patients with periapical lesions for a comparison to a group without periapical lesions. Analyzing sinus abnormalities, they showed a relation between periapical lesions and sinus obliteration. Another aspect is the possible association between sinus obliteration and the time of the year. In contrast to other studies [16–18], the present study showed no seasonal differences in the presence of obliteration of the maxillary sinus.

Velasco-Torres et al. [19] showed a larger sinus volume for dentate patients compared to edentulous and partially edentulous patients. This may be explained through the loss of posterior teeth in the maxilla, leading to the reduction of mechanical stimulation of the maxillary sinus. As a consequence, the sinus could expand (pneumatization) due to increased pressure and ostoclastic activity of the Schneiderian membrane [7, 20–23]. Another factor of influence may be bone resorption following tooth loss [24]. In the present study, however, no significant association between the sinus volume and the state of dentition could be found.

### Patient-level analysis

The results of this study showed a statistically significant smaller mean osseus sinus volume in women compared to men, confirming previous findings [19, 25, 26]. Comparing the bilateral situation, the study showed that both maxillary sinuses (osseus borders) of each participant had similar osseus volumes (mean difference between left and right 0.5 cm^3^), thus confirming previous studies [9, 27–30]. Also confirming other studies [29, 30], the results show no association between the participant’s age and the maxillary sinus volume. This is in contradiction to Velasco-Torres et al., who showed an increase in sinus volume with the patient’s age [19].

A limitation of this study is the analysis of the influence of variable parameters on the dimensions of the sinus, which would have benefitted of a larger study size. Moreover, an examination of data deviation and identification of potential data outliers would have been possible.

## Conclusions

The present study showed the volume software to be a suitable tool for the measurement of the dimensions of the maxillary sinus. The results show that the osseus volume of the maxillary sinus varies on the base of gender and that the obliterated volume varies on the base of a present pathology. No statistically significant association between the patient’s age and the sinus volume or a present sinus pathology, the scan date (winter/autumn) and a sinus pathology, communicating roots and sinus pathologies, or unilateral obliteration and apical radiolucencies could be found.
